# Poliomyelitis amidst COVID-19 pandemic in Pakistan: A perspective

**DOI:** 10.1097/MS9.0000000000000168

**Published:** 2023-02-07

**Authors:** Abhigan B. Shrestha, Muhammad H. Nawaz, Sajina Shrestha, Pashupati Pokharel, Jawad Basit

**Affiliations:** a M Abdur Rahim Medical College, Dinajpur, Bangladesh; b KIST Medical College, Imadol, Patan; c Maharajgunj Medical Campus, Institute of Medicine, Kathmandu, Nepal; d Department of Medicine, Rawalpindi Medical University, Rawalpindi, Punjab, Pakistan

*Dear Editor*,

Poliomyelitis is an acute viral disease caused by a single-stranded RNA poliovirus. This virus belongs to the family *Picornaviridae* and to the *Enterovirus* genus, which is again classified into three serotypes (types 1, 2, and 3). The virus affects mainly children under the age of 5 years. This virus invades the central nervous system and leads to paralysis, even causing death. It is transmitted via the fecal-oral route, especially in contaminated water and food. Being an ancient disease, still, no treatment is available for this lethal virus. The only possibility is its prevention through vaccination^1^. The WHO Region of South East Asia was declared a polio-free zone in March 2014. This accomplishment has saved the lives of millions of children, as more than 80% of the world’s population now lives in polio-free regions^1^. However, some countries, namely Pakistan, Afghanistan, and Nigeria, fall on the darker side of the moon.

Pakistan and Afghanistan together have predominately contributed 85% of the current world’s polio cases. Looking at the curves since the pandemic, it has been surging at its peak. Wild Poliovirus (WPV) cases were 84 and 135 cases of Circulating Vaccine derived Poliovirus (cVDPV2) during 2020, then dipped to only one WPV case and cVDPV2 to eight cases in 2021, and this year already 17 cases of WPV have been noted. These cases are solely reported from the Khyber Pakhtunkhwa Province of Pakistan, with WPV of 15 cases from North Waziristan and two cases from Lakki Marwat district^2^. Compared to previous years, where cases were more scattered in different provinces, including Punjab, Sindh, and Balochistan, to date, cases are only contained in Khyber Pakhtunkhwa^2^. Owing to this surge in cases, the Centers for Disease Control and Prevention has announced an alert level 2 for travelers hailing from Pakistan. A single lifetime booster dose of inactivated polio vaccine has been recommended for those who have received a routine polio vaccine and a full series of vaccinations for unvaccinated ones.

Pakistan, a generally warm country, is plagued by poor socioeconomic and law and order situations. The abundance of slums and poor sanitation, even in rather developed parts of the city, is fertile soil for polio infection. The strain of WPV was even found in the water supply of Gaddap and other parts of Pakistan^3^. Significant environmental challenges, for example, the recent monsoon flooding of Southern Punjab, Central Sindh, parts of Balochistan, and insurgency in the tribal belt neighboring Afghanistan, have displaced the population in temporary overcrowded settlements recently, and in the past. A misconception that taking the vaccine makes the baby sick, and that it contains haram materials, such as pig by-products, has further halted its use^3^,^4^. This makes vaccination in areas outside the writ of the government highly difficult and insecure. Despite polio being a heavily funded program in the west, the grassroots infrastructure faces difficulties due to continuously changing political and law and order situations^5^.

With the ongoing fourth wave of coronavirus disease-2019, more than 1.5 million people have been affected, and the resources and infrastructure are still lacking in many places^6^. The devastation has made the country fragile and futile, and any infectious outbreak could lead to a total health crisis. Adding polio to the list can prove to be the worst nightmare for Pakistan. Adding to this, the monkeypox outbreak has made fear among the people, and already south East Asian nations are lacking much preparedness^7^.

To overcome this threat, health authorities must ensure universal access to proper sanitation and clean water. Misbelieves and misconceptions about polio vaccination among community members should be addressed by tailored propaganda specific to the needs of the local community; finding local public health officials for high-risk areas and providing them with epidemiological training is a pivotal strategy in aiding mass polio vaccination. Intervention of multiple stakeholders, both political and religious, is required at different levels of the health system. Morals of the polio workers should be kept high by consistent wages, promotion opportunities, and job and life security. Integration of polio activities with other preventive health services, especially child health, nutrition, and routine immunization, may address community resistance and increase program reach. Augmenting the healthcare system to replace oral polio vaccine completely with parental polio vaccine as in tropical countries like Pakistan and India, oral polio vaccine has decreased per dose efficacy^8^. Furthermore, the concept of immunizing 80–85% of the population with herd immunity seems practicable in this situation. Along with the rest of the world, Pakistan should also focus on strategies to eradicate this virus once and for all.

## Ethical approval

Not required.

## Patient consent

Not required.

## Sources of funding

None.

## Author contribution

A.B. Shrestha: conceptualization. All authors were involved in various aspects of writing and editing of the manuscript.

## Conflicts of interest disclosure

The authors declare no conflict of interest.

## Research registration unique identifying number (UIN)

Not required.

## Guarantor

Pashupati Pokharel

## Data availability

All data are presented within the manuscript.

## Provenance and peer review

Not commissioned, externally peer-reviewed.
